# HUGO^TM^ RAS System in Robotic-Assisted Radical prostatectomy is highligheted in International Brazilian Journal of Urology

**DOI:** 10.1590/S1677-5538.IBJU.2023.02.01

**Published:** 2023-05-29

**Authors:** Luciano A. Favorito

**Affiliations:** 1 Unidade de Pesquisa Urogenital Universidade do Estado do Rio de Janeiro Rio de Janeiro RJ Brasil Unidade de Pesquisa Urogenital - Universidade do Estado do Rio de Janeiro - Uerj, Rio de Janeiro, RJ, Brasil,; 2 Serviço de Urologia Hospital Federal da Lagoa Rio de Janeiro RJ Brasil Serviço de Urologia, Hospital Federal da Lagoa, Rio de Janeiro, RJ, Brasil

The March-April number of *Int Braz J Urol* is the 21^th^ under my supervision. In this number the Int Braz J Urol presents original contributions with a lot of interesting papers in different fields: Testicular cancer, SARS-CoV-19, Robotic Surgery, Prostate Cancer, Endometriosis, Infection and Profilaxy in Urology, Translational Research, Male Health and Renal stones. The papers came from many different countries such as Brazil, Panama, China and USA, and as usual the editor´s comment highlights some of them. The editor in chief would like to highlight the following works:

Dr. Barros and collegues from Brazil and USA, presented in page 175 ( 1 ) a nice review about the Changes in male sexuality after urologic cancer and concluded that male sexual dysfunction is very common after urologic cancer diagnosis and treatment. Changes in body image and anatomical damage can be associated with impaired masculinity and sexual function, especially after prostate, penile or testicular cancer treatment. Moreover, anxiety, depression, and fear of recurrence have an impact on quality of life and sexual function regardless of the cancer location. Therefore, patients need be counseled about the likely changes in sexual function before treatment of any urological cancer.

Dr. Danilovic and collegues from Brazil, presented in page 184 ( 2 ) an important systematic review about the topic: “the use for one week pre-operative oral antibiotics for percutaneous nephrolithotomy reduce risk of infection” and concluded that one week of prophylactic oral antibiotics based on local bacterial sensi- tivity pattern plus a dose of intravenous antibiotics at the time of surgery in patients undergoing PCNL reduces the risk of infection.

Dr. Liao and collegues from China, presented in page 194 ( 3 ) a interesting comparative study about the topic: “dusting to basketing for renal stones ≤ 2 cm during flexible ureteroscopy” and concluded that dusting has advantages in shortening the operation time and reducing the operation cost, but the lasing time was longer compared with the basketing. Although there is no difference in long-term effect, basketing is superior to dusting in terms of short-term SFR. Moreover, dusting should be avoided in some special cases and basketing a better choice. Both techniques are effective for the treatment of renal stones ≤ 2 cm and choice depends on patient demographic and stone characteristics.

Dr. Silva Filho and collegues from Brazil, presented in page 202 ( 4 ) a original study about the use of dynamic cystoscopy (DC) to optimize preoperative assessment of bladder endometriosis and concluded that dynamic cystoscopy appears to be a highly specific test with lower sensitivity. DC abnormalities are associated with a higher ratio of bladder surgery for the treatment of deep endometriosis, and bladder endometriosis type 2 seems to be associated with a greater ratio (9.72) of partial cystectomy.

Dr. Alfano and collegues from USA, presented in page 211 ( 5 ) the cover paper of this edition, a nice study about robotic surgery. The authors described the experience with the implementation of the HugoTM RAS robot and report the clinical data of patients who underwent Robotic-assisted Radical Prostatectomy and concluded that this preliminary results with safe and feasible procedures performed with HugoTM RAS System robotic platform. The surgeries were successfully executed with acceptable perioperative outcomes, without conversions or major complications. However, as this technology is very recent, further studies with a long-term follow-up are awaited to access postoperative functional and oncological outcomes.

Dr. Hong and collegues from China, presented in page 221 ( 6 ) a interesting study about a Predictive model for urosepsis in patients with Upper Urinary Tract Calculi based on ultrasonography and urinalysis using artificial intelligence learning and concluded that a preliminary screening model for urosepsis based on ultrasound and urinalysis was constructed using ANN. The model could provide risk assessments for urosepsis in patients with upper urinary tract calculi.

Dr. Viana and collegues from Brazil, presented in page 243 ( 7 ) a interesting study about sex with animals (SWA) and sexual infections (STIs) and concluded that SWA practices increase STIs vulnerability. The association between hepatitis B and SWA highlights the importance of educational campaigns and conclusive studies on the topic.

Dr. Andrade and collegues from Brazil, presented in page 233 ( 8 ) a interesting study about the Impact of COVID-19 pandemic on prostate cancer outcomes at an uro-oncology referral center and concluded that there was no delay between diagnosis and treatment at our institution during the COVID-19 pandemic period. No worsening of the prostate cancer features was observed.

The Editor-in-chief expects everyone to enjoy reading.
